# Paradoxical Left Ventricular Hypertrophy by Echocardiogram and Low Voltage ECG: A Key Clue in the Diagnostic Workup of Two Distinct Presentations of Cardiac AL Amyloidosis

**DOI:** 10.7759/cureus.39143

**Published:** 2023-05-17

**Authors:** Oluwaremilekun Tolu-Akinnawo, Kikelola Oyeleye, Timothy Thayer

**Affiliations:** 1 Internal Medicine, Meharry Medical College, Nashville, USA; 2 Cardiology, Vanderbilt University Medical Center, Nashville, USA

**Keywords:** diastolic heart failure, low voltage ecg, paradoxical left ventricular hypertrophy, cardiac amyloidosis, right heart failure, al amyloidosis

## Abstract

Cardiac amyloidosis remains a rare disease caused by the extracellular deposition of abnormal proteins-amyloids in the myocardium. These protein structures in the myocardium are associated with high morbidity and mortality, with prognosis hinging on early detection and treatment. Three main types of cardiac amyloidosis have been identified: light chain (AL), familial or senile (ATTR), and secondary amyloidosis which is associated with chronic inflammation. Cardiac amyloidosis classically presents as diastolic heart failure with symptoms of volume overload low voltage on electrocardiogram (ECG) and echocardiographic features of diastolic dysfunction and paradoxical left ventricular hypertrophy (paradoxical with respect to low voltage on ECG). Early suspicion should trigger additional laboratory and imaging workup to facilitate early detection. Early detection remains critical to prognosis. Herein, we present two patients admitted to a safety-net hospital within one month of each other with distinct presentations yet important, overlapping characteristics that led to the diagnosis of AL amyloidosis in both patients.

## Introduction

Amyloidosis is a rare disease caused by the extracellular deposition of abnormal protein-polysaccharide complexes called amyloids in various organs and tissues, leading to cellular dysfunction, damage, or even death. AL amyloidosis accounts for 9.7-14 cases per 1, 000,000 persons, with at least 12,000 adults currently living with AL amyloidosis in the United States [1]. AL cardiac amyloidosis typically presents as progressive chronic heart failure: dyspnea at rest and exertion, peripheral edema, pleural effusion, and other signs of volume overload, as seen in our patients. Diagnosing AL amyloidosis is complicated and requires high suspicion to trigger appropriate laboratory and imaging studies. A definite diagnosis remains a tissue diagnosis. These cases illustrate typical and atypical presentations of cardiac AL amyloidosis. Cardiac and systemic damage caused by the AL amyloidosis disease process is irreversible; thus, early diagnosis and treatment are critical to prognosis by limiting further amyloid deposition [2].

## Case presentation

Patient 1 - More typical presentation of cardiac AL amyloidosis

A 62-year-old male with no known medical history and who has not visited a physician in several years presented to the ambulatory clinic with complaints of generalized body swelling and shortness of breath for three months. He endorsed dyspnea on exertion, cough productive of clear whitish sputum, weight gain of about 20 pounds (from baseline 187 pounds up to 207.6 pounds) over three months, palpitations, orthopnea, and paroxysmal nocturnal dyspnea. He denied chest pain and syncope or near syncopal symptoms. The patient smoked one cigar daily for ten years and denies alcohol intake or use of illicit substances. On presentation, his BP was 127/74mmHg, and his body mass index (BMI) was 26.9. Labs obtained showed an elevated Pro-B-type natriuretic peptide (Pro-BNP) of 8356 pg/mL with normal high-sensitivity troponin and thyroid function tests. Complete blood counts and comprehensive metabolic panel were normal. No proteinuria was noted on urinalysis. Laboratory findings comparing patients 1 and 2 are indicated in Table 1 below.

**Table 1:** Laboratory findings comparing Patient 1 and Patient 2 Pro-BNP: Pro B-Type Natriuretic Peptide; ALT: Alanine Transaminase; AST: Aspartate Aminotransferase; UA: Urinalysis

Laboratory findings	Patient 1	Patient 2	Reference values
Pro-BNP	8356 pg/ml	2890 pg/ml	< 125 pg/ml
ALT	36 U/L	173 U/L	12-78 U/L
AST	30 U/L	112 U/L	15-37 U/L
Troponin	44 pg/mL	3203 ng/ml	4-59 pg/ml
UA	Normal	1+ RBC	Undetectable
Lambda-free light chains	256.30 mg/L	219 mg/L	5.71-26.30 mg/L
Kappa-free light chains	8.09 mg/L	16.98 mg/L	3.30-19.40 mg/L
Kappa/Lambda	0.03	0.08	0.26-1.65

**Figure 1:**
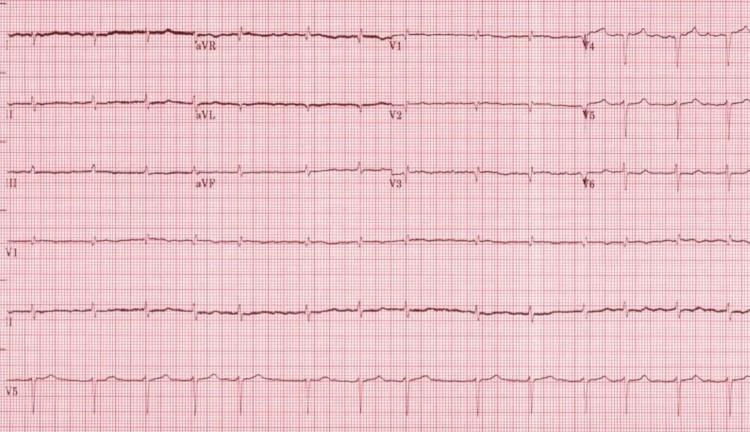
ECG showed low voltage QRS (limb leads), atrial fibrillation, and incomplete right bundle branch block ECG: Electrocardiogram

Electrocardiogram (ECG) showed low voltage QRS (limb leads), atrial fibrillation, and incomplete right bundle branch block with a ventricular rate of 86 beats per minute (Figure 1).

**Figure 2:**
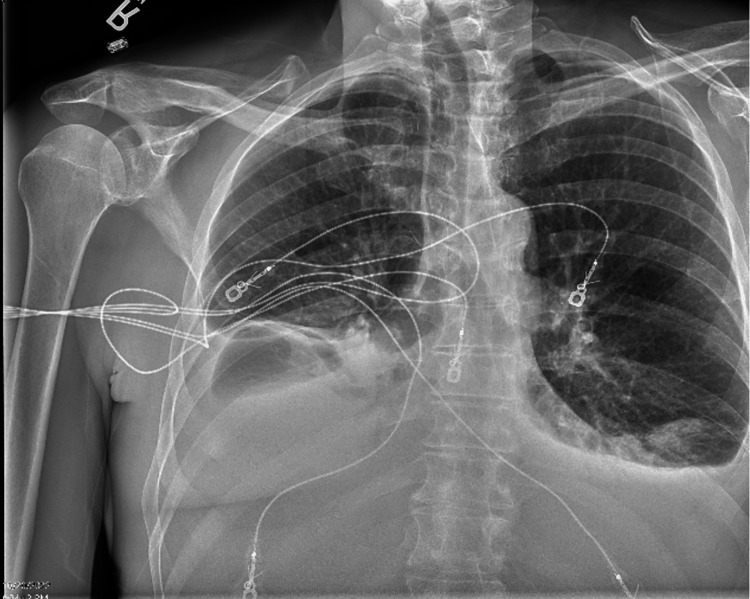
Chest X-ray with bibasilar consolidations with bilateral pleural effusions right greater than the left

Chest X-ray revealed bibasilar consolidations with bilateral pleural effusions right greater than the left (Figure 2).

**Figure 3:**
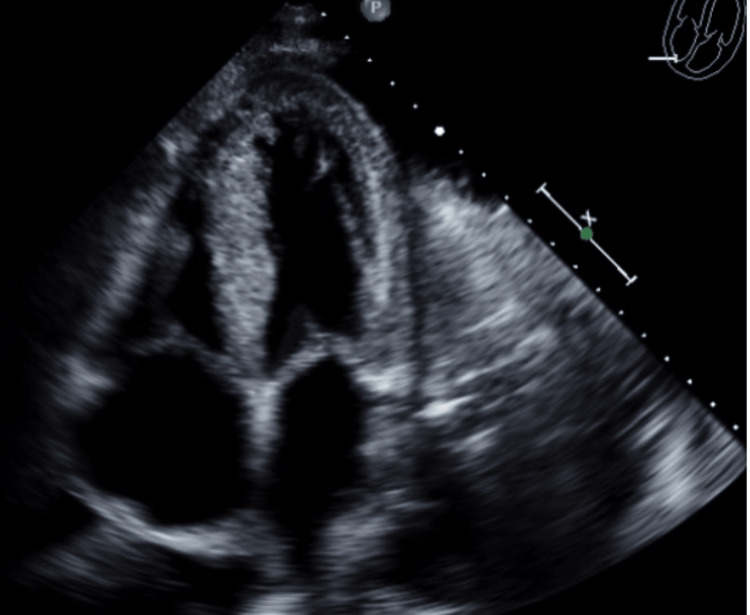
Transthoracic echocardiogram (2D ECHO) done significant for moderate-to-severe bi-ventricular hypertrophy, and systolic function (LVEF >65%), indeterminant diastolic function due to underlying atrial fibrillation LVEF: Left ventricular ejection fraction

A transthoracic echocardiogram (2D ECHO) showed moderate-to-severe biventricular hypertrophy, normal left ventricular (LV) cavity size, and systolic function (LVEF >65%), indeterminant diastolic function in the setting of atrial fibrillation (Figure 3). The right ventricular filling pressure was also moderately reduced with a tricuspid annular plane systolic excursion (TAPSE) of 9mm (normal > 16mm), the left atrial pressure estimate is elevated with a mean E/E of 21 (normal < 15), elevated right atrial pressure at 15mmHg (normal 2-6mmHg), with interatrial septal thickening and mild mitral regurgitation otherwise no other valvular abnormalities. There was also trivial pericardial effusion and large pleural effusion noted.

He was started on IV furosemide and acetazolamide for diuresis [3]. Dapagliflozin was started due to its benefit in patients with heart failure with preserved ejection fraction [4]. Due to concerns for restrictive cardiomyopathy, urine protein electrophoresis, serum protein electrophoresis, serum-free light chains, and iron studies were obtained. Results were notable for elevated lambda quantitative free light chains of 256.3 mg/L with normal Kappa levels of 8.09 mg/L and a decreased Kappa/Lambda ratio of 0.03. Serum protein electrophoresis showed decreased albumin region, suspicious broadening of the complement component in the beta region, and hypogammaglobulinemia with a suggestion for immunofixation to rule out a gammopathy if clinically indicated. Ferritin was 102 ng/mL (ref: 12-300 ng/mL), which ruled out hemochromatosis.

Nutrition/dietary consulted to educate the patient on a low-sodium diet. Regarding the new diagnosis of atrial fibrillation, his CHADsVasc score was 1, which corresponds to an estimated annual risk of stroke of approximately 2.8%; however, the CHADsVasc score is known to grossly underestimate stroke risk in amyloid cardiomyopathy, with estimated intracardiac thrombus rates as high as approximately 33% in cross-sectional studies [5]. Thus, he was started on apixaban to reduce stroke risk. The patient was spontaneously rate controlled. His symptoms of volume overload resolved with diuresis, and he was discharged home on apixaban, furosemide, and acetazolamide with plans to follow up closely at an amyloid-focused hematology clinic and cardiology clinic.

The patient was admitted with recurrent heart failure exacerbation a month after discharge. He underwent a fat pad biopsy that was positive for amyloidosis. Samples sent for flow cytometry confirmed the Lambda AL amyloid protein subtype. A cardiac MRI obtained was consistent with cardiac amyloidosis. A skeletal survey was negative for lytic or blastic lesions. He underwent a bone marrow biopsy to assess for underlying multiple myeloma associated with AL amyloidosis. He was found to have 10-20% plasma cells and a 1q21 gain on Fluorescence in-situ hybridization (FISH) analysis consistent with a plasma cell neoplasm. The patient was initiated on chemotherapy with daratumumab-hyaluronidase, cyclophosphamide, and dexamethasone shortly before the preparation of this manuscript.

Patient 2 - More atypical presentation of cardiac AL amyloidosis

The patient is a 63-year-old with no significant past medical history who presented to the emergency room complaining of worsening shortness of breath for one week. The patient endorsed associated orthopnea, paroxysmal nocturnal dyspnea, and leg swelling, starting about 4-5 days before presentation. He also reported unintentional weight gain of about 20 pounds (222 pounds up to 242.5 pounds) three months before admission. The patient denied chest pain/discomfort, palpitations, diaphoresis, or syncopal episodes.

Vital signs at presentation were significant for elevated blood pressure of 166/97mmHg, saturating at 94% on room air. BMI was 33.2. The patient denies tobacco smoking, significant alcohol intake, or use of illicit substances. Labs were significant for essentially normal complete cell blood counts, normal basic metabolic panel, elevated alanine transaminase (ALT) 173 U/L, elevated aminotransferase (AST) 112 U/L, elevated high-sensitivity troponin of 3203 pg/mL, elevated Pro-B-type natriuretic peptide of 2890 pg/mL, borderline elevated thyroid stimulating hormone of 4.620 uU/mL (normal: 0.358-3.740 uU/mL). Urinalysis was positive for 1+ blood and was negative for protein. Iron studies were within normal limits.

**Figure 4:**
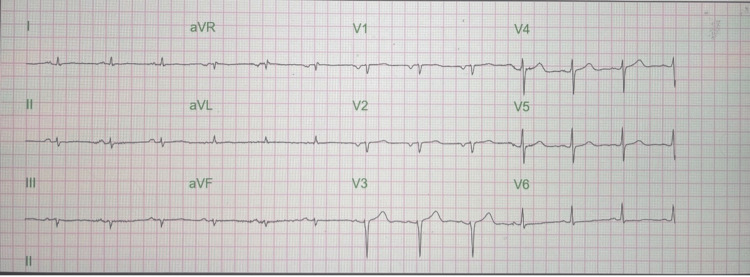
ECG significant for low voltage QRS (limb leads) and left atrial enlargement ECG: Electrocardiogram

ECG revealed low voltage QRS (limb leads), normal sinus rhythm, and left atrial enlargement (Figure 4).

**Figure 5:**
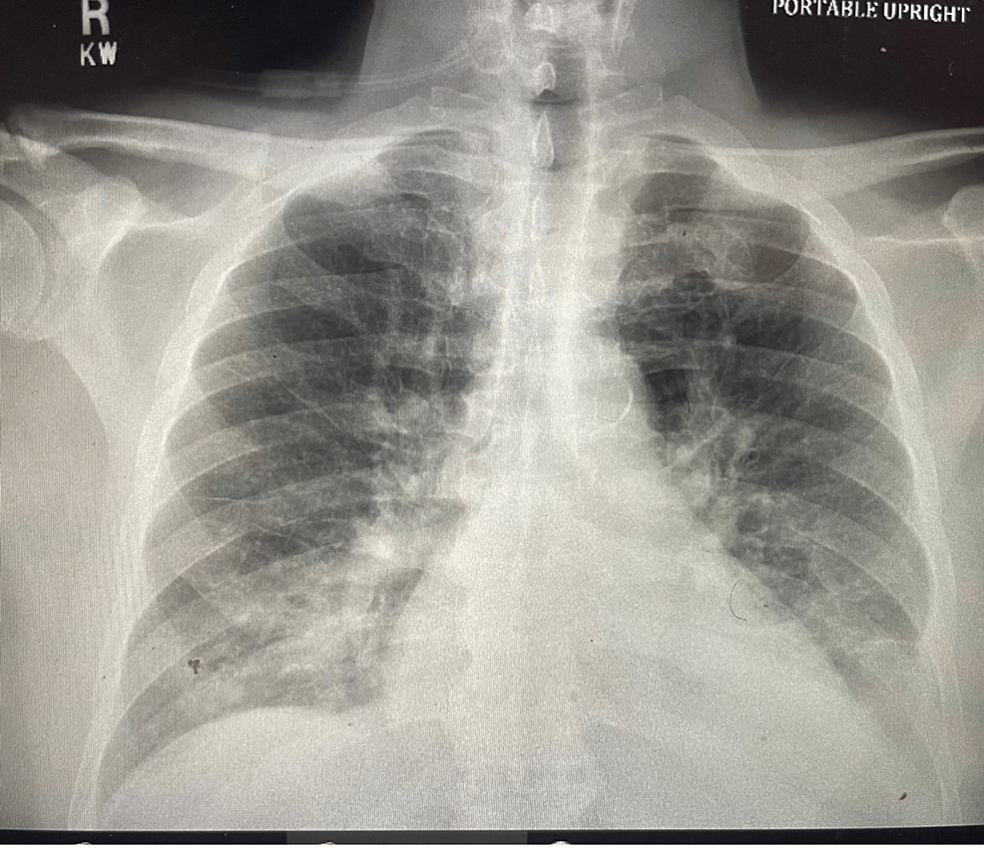
Chest X-ray significant for cardiomegaly with features of congestive heart failure

Chest X-ray revealed cardiomegaly with features of congestive heart failure (Figure 5).

**Figure 6:**
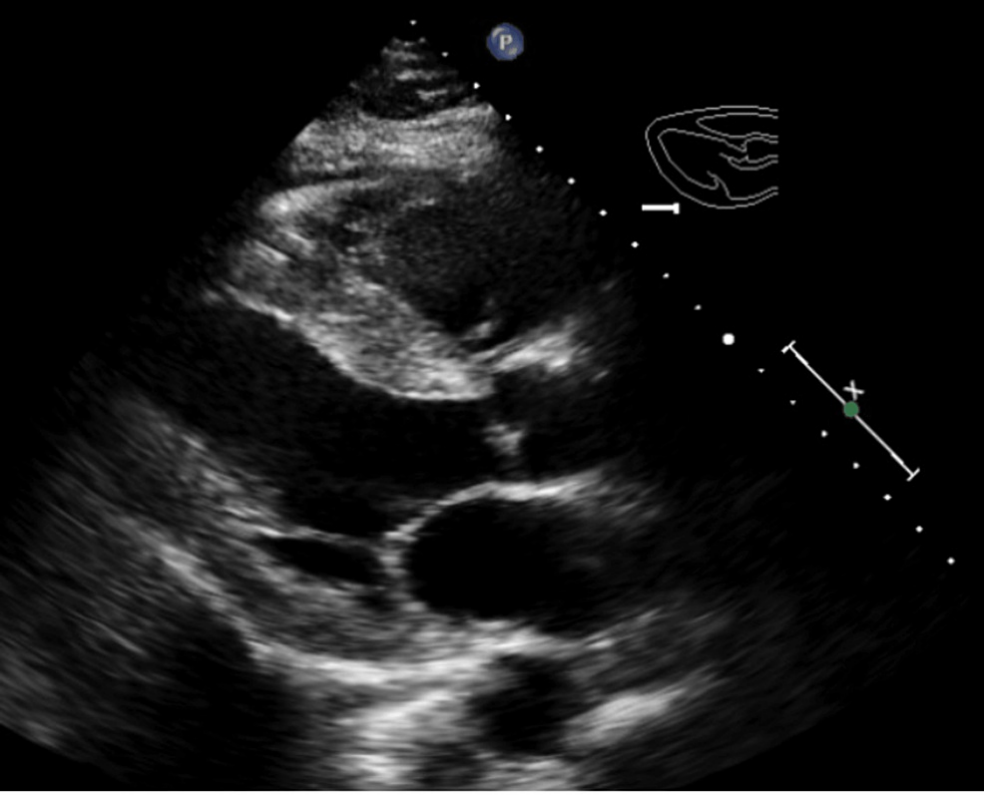
Transthoracic echocardiography revealed severe global hypokinesis and systolic dysfunction with an estimated left ventricular ejection fraction of 25-30%, mild concentric left ventricular hypertrophy, grade one diastolic dysfunction, and mild right ventricular dysfunction

Transthoracic echocardiography (2D ECHO) revealed severe global hypokinesis and systolic dysfunction with an estimated left ventricular ejection fraction of 25-30%, mild concentric left ventricular hypertrophy, grade one diastolic dysfunction, with normal estimated resting left atrial pressure, normal right atrial pressure, no valvular abnormalities, and no pericardial effusion was noted (Figure 6).

The patient was started on intravenous diuresis and guideline-directed medical therapy for heart failure: Carvedilol, dapagliflozin, Entresto, and spironolactone. He was also started on aspirin/atorvastatin therapy. For new-onset heart failure, right and left heart catheterizations were done, which revealed non-obstructive coronary artery disease with an estimated 45% stenosis of the left anterior descending artery. Hemodynamic findings on right heart catheterization (post-diuresis) were notable for normal filling pressures and severely reduced cardiac index (CI) (1.7 L/min/m2 by Fick and 1.9 L/min/m2 by thermodilution; ref: 2.5-4 L/min/m2). Other hemodynamic findings are pulmonary artery (PA) pressure of 31/9/16 mmHg (normal: 25/10/15), pulmonary capillary wedge pressure (PCWP) of 4 mmHg (normal: 4-12 mmHg), and pulmonary vascular resistance (PVR) of 260 dynes/sec/cm5 (normal: 37-250 dynes/sec/cm5). The patient noted significant interval improvement in clinical symptoms and was discharged to follow up with his primary care provider and cardiology clinic.

At follow-up at the cardiology clinic, additional workups were performed due to suspicion of infiltrative cardiomyopathy due to paradoxical left ventricular hypertrophy on 2D ECHO and low voltage on ECG. Serum protein electrophoresis testing was significant for elevated lambda-free light chains of 219 mg/L and a decreased kappa/lambda ratio of 0.08. The patient was again volume overloaded, so he was re-admitted for IV diuresis and accelerated workup of suspected AL amyloidosis. A cardiac MRI revealed biventricular failure (LVEF 29% and RVEF 21%) and “findings consistent with a diagnosis of cardiac amyloidosis.” A pyrophosphate scan was negative for transthyretin-related cardiac amyloidosis. A skeletal survey showed no radiographic evidence of multiple myeloma. He underwent an initial bone marrow biopsy that was nondiagnostic, and a repeat bone marrow biopsy was negative for amyloid but positive for plasma cell neoplasm (10-15% plasma cells, FISH neg). Right heart biopsy for definitive diagnosis confirmed lambda AL amyloidosis.

## Discussion

These two patients' cases illustrate both the quintessential findings that should trigger a workup for AL amyloidosis as well as how cardiac AL amyloidosis presentations may vary. The combination of low voltage on ECG and paradoxical left ventricular hypertrophy by echocardiogram was found in both patients. It should be a “red flag” for potential cardiac amyloidosis for clinicians caring for individuals with heart failure. Contrasting these two cases, we see some patients with preserved ejection fraction (patient 1) and atrial fibrillation (which precludes assessment of diastolic function). In contrast, others present with reduced ejection fraction and sinus rhythm (patient 2). The second case is a cautionary tale as this patient was not worked up for AL amyloidosis during his index hospitalization for heart failure. It seems likely that his presentation with reduced ejection fraction and hypertension did not trigger suspicion for amyloidosis despite the low voltage ECG.

Amyloidosis is an infrequent, multi-organ disease characterized by the extracellular deposition of fibrils in any body organ, most commonly the heart. The amyloid fibrils are rigid, proteolytic-resistant structures, usually less than 10nm in diameter, with a typical apple-birefringence on Congo-red staining under polarized light microscopy [6]. Three main types of cardiac amyloidosis have been identified: light chain (AL), familial or senile (ATTR), and secondary amyloidosis- which is associated with chronic inflammation [7]. However, 95% of cardiac amyloidosis cases are secondary to transthyretin amyloidosis (ATTR) and light chain amyloidosis (AL) [6]. AL is a clonal plasma cell disorder secondary to increased production and misfolding of antibody light chain fragments [8]. The median age of presentation is 63; however, patients in their 30’s and 40’s can also present in this case [9, 10]. AL is more aggressive than ATTR, with a median untreated survival of fewer than six months in patients presenting with heart failure. This further illustrates the need for early detection and treatment, as seen in our patient [8]. While ATTR is less aggressive than AL, it is still a progressive disorder with significantly limited survival and quality of life [8].

The deposition of fibrils leads to the thickening of both ventricles, making them stiff and poorly compliant, resulting in progressive diastolic filling abnormalities and, occasionally, systolic dysfunction. AL amyloid deposition is usually subendocardial and diffuse, while ATTR patterns can be patchy areas of transmural involvement. Also, the atria are universally involved with interatrial septal thickening leading to poor atrial function and increased rates of atrial fibrillation, as seen in patient 1 [8]. The conduction system could also be affected, leading to varying degrees of heart block that may require pacemaker implantation [11]. Valvular involvement also leads to an increased risk of regurgitation. Pericardial involvement can lead to small and rarely large pericardial effusions, as seen in patient 1 (pericardial effusion absent in patient 2). Less commonly, the involvement of coronary vessels can lead to ischemia and angina. Other symptoms to increase suspicion are macroglossia, periorbital purpura, proteinuria (usually a nephrotic range), jaw claudication, diarrhea, or weight loss [12]. Neurologic symptoms, such as carpal tunnel syndrome or peripheral neuropathy, may also be the harbingers of amyloidosis [12]. The presence of non-cardiac symptoms highlights the systemic nature of the disease.

Routine laboratory findings such as elevated N-terminal pro-b-type natriuretic peptide and elevated troponins are non-specific; however, they should raise suspicion for underlying cardiac dysfunction. If amyloidosis is suspected, a workup for monoclonal antibodies should be initiated. Although helpful, serum and urine protein electrophoresis are insensitive tests to detect AL amyloidosis. Serum-free light chain assays that measure free kappa and lambda light chain levels and, most pertinently, the ratio of kappa to lambda is much more sensitive [8]. This test should be measured alongside immunofixation of the serum and urine.

ECG findings in cardiac amyloidosis typically revealed low QRS voltage, a finding more commonly seen in AL amyloidosis versus ATTR, as seen in both of our patients [13]. A pseudo infarction pattern (QS waves in consecutive leads) and conduction delays in the form of blocks are also typical [13]. Other arrhythmias that could be seen besides atrial fibrillation (seen in patient 1) are atrial flutter and ventricular tachycardia [13]. The characteristic features of cardiac amyloidosis on echocardiography are ventricular thickening with a myocardial ‘speckled’ appearance, reduced left ventricular volume, enlarged atria, and restrictive diastolic pattern [13]. A total of 28% of cases of cardiac amyloidosis present with reduced ejection fraction, highlighting the importance of considering cardiac amyloidosis in the workup of patients with heart failure with reduced ejection fraction, as seen in patient 2 [14].

A cardiac MRI is another non-invasive imaging method for detecting cardiac amyloidosis when combined with gadolinium enhancement, boasting a sensitivity rate of 87% [11, 15]. The use of cardiac MRI is, however, limited in patients with grossly impaired renal function, availability of tests, and cost. The gold standard for diagnosing amyloidosis remains histological detection, typically through biopsy [13]. Fine-needle aspiration of the abdominal fat is a standard procedure that is positive for amyloid deposits in about 70% of patients with AL amyloidosis.

Early detection and treatment are crucial to survival since the median survival of untreated cardiac AL is six months [13]. The management of AL amyloidosis is focused on volume optimization and preventing the progression of amyloid deposition. Diuresis remains the cornerstone of symptom management [11]. Angiotensin-converting enzyme inhibitors (ACEIs) and Angiotensin II receptor blockers (ARBs) are poorly tolerated in patients with AL amyloidosis due to the risk of hypotension, even with small doses [11]. Captopril is preferred if a RAAS blockade is to be started due to its shorter duration of action and must be gradually introduced [11]. Clinical deterioration has, however, been identified when cardiac amyloidosis is treated with calcium channel blockers or beta-adrenergic blockers due to the increased risk of orthostatic hypotension and obliteration of sympathetic input in these patients [11]. The beta-blockade, in particular, is not well tolerated in cardiac amyloidosis as patients tend to be heart-rate dependent for cardiac output in the setting of noncompliant ventricles with relatively fixed stroke volumes. Digoxin is also typically avoided due to the increased risk of toxicity as digoxin is recognized to bind tightly to cardiac amyloid fibers and may increase the risk for cardiac digitalis toxicity [16]. It is prudent to start all patients with AL amyloidosis with atrial fibrillation on anticoagulation due to a high annual risk of thromboembolism, as we did in patient 1 [17]. AL management includes oral chemotherapy (greatest survival in patients without cardiac involvement) or high-dose chemotherapy (melphalan- 40-60% remission) with autologous stem cell transplantation [18]. Advanced disease may render patients unfit for high-dose chemotherapy or transplant, hence the importance of early detection and treatment. Without other organ involvement, cardiac transplantation may be an option for potentially curative therapy after definitive treatment of the plasma cell disorder.

## Conclusions

AL cardiac amyloidosis is an infrequent but life-threatening disease that requires a high index of suspicion due to its overlap symptoms with other cardiac disorders. Abnormal laboratory findings of elevated protein gap, pro-BNP, and troponin in patients presenting with signs and symptoms of heart failure should increase suspicion. A key “red flag” to trigger a serologic workup for AL amyloid is the ECG finding of low QRS with paradoxical left ventricular hypertrophy on echocardiogram. Early detection and treatment are crucial to survival, as patients with advanced diseases may be unable to tolerate treatment. This article provides information that will help providers with early suspicion and the timely diagnosis of AL amyloidosis.
